# The effect of omega3 fatty acid supplementation on PPARγ and UCP2 expressions, resting energy expenditure, and appetite in athletes

**DOI:** 10.1186/s13102-021-00266-4

**Published:** 2021-05-08

**Authors:** Sara Moradi, Mohamadreza Alivand, Yaser KhajeBishak, Mohamad AsghariJafarabadi, Maedeh Alipour, Philip D. Chilibeck, Beitullah Alipour

**Affiliations:** 1 Student Research Committee, Tabriz University of Medical Sciences, Tabriz, Iran; 2 Research Center, Faculty of Nutrition and Food Sciences, Tabriz University of Medical Sciences, Tabriz, Iran; 3 Department of Medical Genetics, Faculty of Medicine, Tabriz University of Medical Sciences, Tabriz, Iran; 4 Department of Nutrition, Maragheh University of Medical Sciences, Maragheh, Iran; 5 Department of Statistics and Epidemiology, Faculty of Health, Tabriz University of Medical Sciences, Tabriz, Iran; 6 Medical Student, Faculty of Medicine, Tabriz University of Medical Sciences, Tabriz, Iran; 7 College of Kinesiology, University of Saskatchewan, Saskatoon, SK Canada; 8 Department of Community Nutrition, Faculty of Nutrition, Tabriz University of Medical Sciences, Tabriz, Iran

**Keywords:** Omega3, PPARγ, UCP2, REE, Appetite

## Abstract

**Background:**

Omega3 fatty acids as a ligand of energy-related genes, have a role in metabolism, and energy expenditure. These effects are due to changes in the expression of peroxisome proliferator-activated receptor-gamma (PPARγ) and uncoupling protein2 (UCP2). This study evaluated the effect of omega3 supplements on PPARγ mRNA expression and UCP2 mRNA expression and protein levels, as regulators of energy metabolism, resting energy expenditure (REE), and appetite in athletes.

**Methods:**

In a 3-week double-blind RCT in Tabriz, Iran, in 2019, 36 male athletes, age 21.86 (±3.15) y with 16.17 (±5.96)% body fat were randomized to either an intervention (2000 mg/day omega3; EPA: 360, DHA: 240) or placebo (2000 mg/day edible paraffin) groups. Appetite and REE were assessed before and after the intervention. PPARγ and UCP2 mRNA expression and UCP2 protein levels in blood were evaluated by standard methods.

**Results:**

Results showed PPARγ mRNA levels, and UCP2 mRNA and protein levels increased in omega3 group (*p* < 0.05), as did REE (p < 0.05). Also, differences in the sensation of hunger or satiety were significant (p < 0.05).

**Conclusions:**

Our findings showed that omega3 supplementation leads to the up-regulation of PPARγ and UCP2 expressions as the indicators of metabolism in healthy athletes.

## Background

Body composition is important for athletes in various types of sports because performance is affected by the ratio of fat mass (FM) and fat-free mass (FFM) [1–3]. For many athletes reducing body fat, it is associated with improved performance, such as material arts, and handball [4, 5]. Although, many factors change body composition, most of these factors are related to the body’s metabolism [6, 7]. Nutrition and physical activity are important lifestyle factors that affect metabolic markers associated with energy metabolism [8]. Studies showed nutrients such as omega3 fatty acids have potential to reduce FM accumulation particularly visceral fat [9–17]. The effect of omega3 fatty acids on weight (fat) reduction is due to increased fat oxidation, and energy expenditure [10]. Omega3 alters multiple signaling pathways, including those related to p21, p53, nuclear f4ractor κB, and STAT [18, 19].

Omega3s, which are essential for humans, mainly include eicosapentaenoic acid (EPA; 20:5), and docosahexaenoic acid (DHA; 22:6) from marine foods and supplements [20–25]. It is recommended that adults consume 500 mg/day of EPA and DHA by two servings of oily fish such as salmon, herring, and mackerel per week [26]. But the intake of omega3 from foods contributes to the provision of only a small amount of daily requirements [27]. Thus, the American Heart Association recommends if the adults do not consume enough fish, they should consume omega3 supplements [13].

On the other hand, omega3 could act as a ligand for peroxisome proliferator-activated receptor gamma (PPARγ) [8, 28]. PPARγ is a steroid nuclear transcription factor that regulates the expression of many genes to modulate energy metabolism, cell differentiation, and apoptosis [29–31].. PPARγ is mainly expressed in adipose tissue and is directly related to adipocytes [32]. An animal study showed omega3 fatty acids decrease fat in adipose tissue through the expression of PPARγ [30]. However, few studies on humans have been conducted. Although, the results of these studies imply that PPARγ gene expression partially controlled by nutritional regulation [10, 30]. PPARγ activates the expression of other genes involved in energy metabolism, such as uncoupling protein 2 (UCP2). UCP2 is located in the inner mitochondrial membrane and promotes leakage of protons. Changes in the proton gradient affects ATP production. The expression of the UCP2 decreased in cardiovascular diseases [33–36]. But the effect of omega3 supplementation on UCP2 mRNA expression and UCP2 protein is controversial [37–40].

Interestingly, UCP2 up-regulates PPARγ expression, and their effect on metabolism may be synergistic [41, 42]. As the PPARγ and UCP2 genes expression are increased, the production of various hormones and neurotransmitters are enhanced. This process increases the proteins that affect metabolism, and resting energy expenditure. Also, changes in the expression of these genes could change blood lipids, fat mass, and appetite [43–46]. For example, increasing PPARγ expression suppresses ghrelin and thus reduces appetite [47]. Another study showed UCP2 suppresses appetite by modulating ghrelin expression [48]. As a change in appetite could alter the food intake, it might affect fat accumulation and metabolism [47, 49].

So far no study has directly examined the relationship between omega3 supplementation, expression of metabolism-related genes, and mitochondrial proteins. Considering that maintenance of optimal body composition is important for many athletes, this study aimed to determine the effect of omega3 supplementation on the expression of the PPARγ, UCP2, and the concentration of UCP2 protein in the blood, resting energy expenditure, and appetite in healthy athletes.

## Method

### Subjects and study design

This was a randomized double-blind placebo-controlled trial involving 36 athlete men. The duration of the project was three weeks. Following a public announcement of the study, volunteers who willing to participate were recruited from public and private gyms, teams, stadiums, councils, and departments of sports, departments of physical education, and the sports medicine board in Tabriz, Iran. After being given a full explanation of the study procedures, participants who agreed to enroll in the study signed a statement of informed consent before the commencement of baseline data collection. The study procedure and the informed consent form were approved by the ethics committee of the medical university of Tabriz (IR.TBZMED.REC.1398.782) in October 2019, and the procedures were performed in accordance with relevant guidelines. The trial was registered at the Iranian registry of the clinical trial website (www.irct.ir) as IRCT20190625044008N1 (https://en.irct.ir/trial/43332), registered at (19/12/2019).

### Inclusion/exclusion criteria

The inclusion criteria were: 1) athlete volunteers who were ranked nationally or players of a professional sports league (football, volleyball, swimming, etc.); 2) age range of 20 to 30 years; 3) BMI between 18.5 to 25 kg/m^2^; 4) avoidance of any dietary supplements, vitamins, minerals, and protein powders at least six months before and throughout the intervention; 5) Not having a history of coagulopathy blood disease, liver damage, kidney disease, pancreatitis, inflammatory diseases, diabetes, cancer, thyroid disorders,, and heart disease; 6) not smoking. The exclusion criteria were: 1) allergic response to the omega3; 2) unwillingness for cooperation; 3) any major change in diet, duration, level, or type of physical activity and regular lifestyle;

### Sample size

The sample size was estimated by considering the expected differences (d) between the two studied groups for one of the main outcomes (REE was used from a previous clinical trial [44]). We calculated the sample size as follows: d = 3.77 Z1-α/2 = 1.96 α = 0.05 1-β = 0.90 Z1-β = 1.282

$$ \mathrm{n}=\raisebox{1ex}{$\left({\left(\left(z1-\frac{\alpha }{2}\right)\left(z1-\beta \right)\right)}^2\ast {\left( SD1+ SD2\right)}^2\right)$}\!\left/ \!\raisebox{-1ex}{${(d)}^2$}\right. $$

According to the equation above, the sample size was calculated as 14 in each group and we selected 18 in each group, to account for a possible 30% loss to follow-up or discontinued intervention.

### Randomization, blinding, and study procedures

Participants who met the eligibility criteria were randomly assigned to the omega3 (*n* = 18) and placebo (n = 18) groups. For randomization, a blinded colleague who was not involved in any of the study stages randomly divided the participants into the intervention and placebo groups (1:1) by using RAS (Random Allocation Software). Omega 3 or placebo containers with identical labeling and they were similar in terms of color, shape, and size. Gelatin capsule supplements and placebo were stored at room temperature. The adequate intake of omega3 for men between the ages of 19–50 years is 1600 mg per day. In similar previous studies, the dose of omega3 supplementation ranged from 200 mg to 6 g per day, and by considering the low amount of omega3 in of the Iranian diet, an effective dose at 2 g per day was intended for this study [50–53]. Participants were stratified into two groups:

1. The Omega 3 group receiving supplements of two Omega 3 soft gel capsules per day (Zahravi Pharmaceutical Co, Tabriz, Iran, consists of 240 mg of DHA, 360 mg EPA).
2. The placebo group receiving placebo two soft gel capsules per day, each capsule containing one g of edible paraffin oil (provided by Zahravi Pharmaceutical, Co., Tabriz, Iran).

Participants were asked to return bottles of supplement and the compliance of participants was evaluated by counting the number of unconsumed capsules at the end of the intervention. None of the participants who completed the trial had compliance less than 90%; therefore, no participants were excluded for inadequate compliance. Participants were advised to maintain their regular diet and level of physical activity during the study. Participants were contacted weekly to track any problems or adverse events, reminded to take their supplements, and to evaluate whether diet or physical activity had changed. None of the participants were excluded because of substantial changes in diet or physical activity. Adverse events were also tracked for a week after the end of the intervention.

### Assessment of physical activity

Physical activity levels were estimated with the global physical activity questionnaire (GPAQ) [54]. A trained researcher filled out the questionnaire for each participant via face-to-face interview. The validity and reliability of the GPAQ have been previously confirmed [55]. Data were processed according to guidelines for analysis of the GPAQ and total metabolic equivalents score (MET-minutes/week) was calculated, with participants categorized as high (≥3000 METs), moderate and low (< 3000 METs) levels of activity.

### Assessment of appetite

A 10 cm visual analog scale (VAS) questionnaire (with six items: hunger, satisfaction, desire to eat, desire to eat sweet, desire to eat salty, desire to eat fatty) were completed in the morning after fasting and after giving blood samples. The validity and reliability of this questionnaire were previously reported [50].

### Anthropometric and blood pressure measurements

Anthropometric parameters were measured in a fasting state. The measurements were performed by a trained nutritionist. Body mass was measured to the nearest 0.1 kg with minimal clothing and without shoes using a digital Seca Beam Balance (Seca, Hamburg, Germany). Height was measured to the nearest 0.1 cm without footwear using a stadiometer in a standing position (Seca, Hamburg, Germany) Body composition was assessed by bioelectrical impedance analysis using the Tanita MC-780 S MA (Amsterdam, the Netherlands). Blood pressure was measured in a comfortable sitting position on the left arm using an aneroid sphygmomanometer and stethoscope after at least five-minutes rest on two occasions and the mean of the two was taken as the individual’s blood pressure.

### REE measurements

Resting energy expenditure (REE), and maximum oxygen consumption (VO2 max) ml/min was measured by indirect calorimetry using the Fitmate Pro (Cosmed, Rome, Italy), which has good validity and reliability for assessment of REE in adults [56]. Energy expended during human performance can be measured by the volume of oxygen witch can consume while exercising at maximum capacity. VO2 max is the maximum rate of oxygen consumption.

### Blood preparation for protein analysis

5 ml of whole blood was collected from all participants after 10–12 h of overnight fasting. 1 ml was transferred to a sterile microtube without any anticoagulant, centrifuged at 3000 RPM for 5 min, and the separated serum stored at − 70 °C until UCP2 was measured. The enzyme-linked immunosorbent assay (ELISA) method was applied to measure serum UCP2 protein by commercial kits (Shanghai Crystal Day Biotech Co., LTD, China) (Intra-assay Precision (Precision within an assay): CV% < 8%; Inter-assay Precision (Precision between assays): CV < 10).

### Gene expression assessment

For measurement of gene expression, the remaining 4 ml of whole blood was used for the isolation of peripheral blood mononuclear cells (PBMCs) immediately after collection in tubes containing EDTA with anticoagulant (Vacutainer K2E). PBMCs were isolated by Ficoll Hypaque density-gradient centrifugation (Miltenyi Biotech GmbH, Bergisch Gladbach, Germany). Total RNA purification was conducted by using the Ambion trizol reagent (Thermo Fisher Scientific), according to the manufacturer’s protocol. The quantity and quality of the RNA was assessed by using NanoDrop Spectrophotometry (NanoDrop OneC; Thermo Fisher Scientific). Then, complementary DNA (cDNA) synthesis was done according to the manufacturer’s protocol (ExcelRT One-Step RT-PCR Kit; smobio).

The integrity of the total RNA of the participants’ samples were assessed by gel electrophoresis (on a 1% agarose gel). For Real-time polymerase chain reaction, the PPARγ and UCP2 gene sequences were acquired from the National Center for Biotechnology Information (NCBI) and Ensembl (http://asia.ensembl.org/) databases. The OLIGO7 Software (Molecular Biology Insights, Inc., Cascade, CO) was used for designing the primer pairs PPARγ and UCP2 of mRNA sequence. Table 1 shows the PPARγ, UCP2, and glyceraldehyde-3-phosphate dehydrogenase (GAPDH) sequences of primers for the polymerase chain reaction. The level of PPARγ and UCP2 mRNA were examined using SYBR Green Master Mix (RealQ Plus 2x Master Mix Green, ampliqon, Denmark). The primer sequences for the human genes of PPARγ, UCP2, and GAPDH (as a housekeeping gene) were evaluated, and the data normalized to GAPDH mRNA expression by using the ΔΔCT comparative method. The fold changes of the PPARγ and UCP2 mRNA were calculated by using the REST Software as the relative expression of post-intervention/placebo [57].

**Table 1:** The sequences of PPARγ, UCP2, and GAPDH primers for polymerase chain reaction

Name	Base pair	Sequences (5′ 3′)
UCP2 (Forward)	18	GGCTGGAGGTGGTCGGAG
UCP2 (Reverse)	22	CAGAAGTGAAGTGGCAAGGGAG
PPARγ (Forward)	20	CTTCCATTACGGAGAGATCC
PPARγ (Reverse)	19	AAAGAAGCCAACACTAAAC
GAPDH (Forward)	20	CTGACTTCAACAGCGACACC
GAPDH (Reverse)	23	CGTTGTCATACCAGGAAATGAGC

*PPARY* Peroxisome proliferator-activated receptor-gamma, *UCP2* Mitochondrial uncoupling protein 2, *GAPDH* glyceraldehyde 3 phosphate dehydrogenase

### Statistical analysis

The analyses were performed using STATA version 16 (StataCorp, College Station, TX, USA). Normality was checked by the Kolmogorov- Smirnov and Shapiro-Wilk test. Data were expressed as mean (SD) and frequency (percent) for categorical variables. Between-group comparisons of baseline measures and demographic variables were done by independent t-test, Mann-Whitney U test, and/or Chi-square test where appropriate. For within-group comparisons analysis of covariance (ANCOVA) and Wilcoxon sign-rank test were used, respectively, before and after intervention. In all analyses, *P* values less than 0.05 were considered as significant.

## Results

**Fig. 1:**
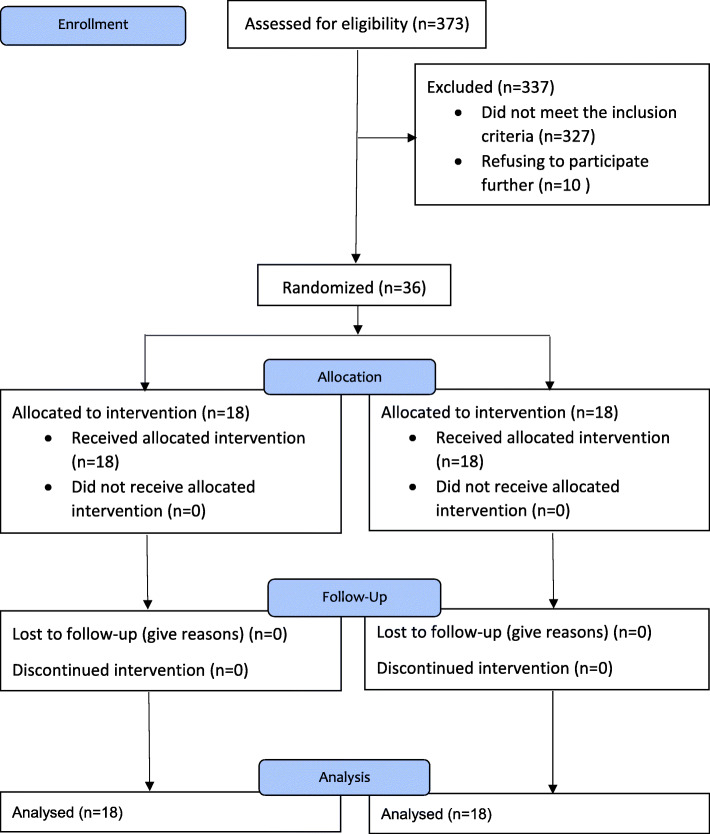
Flow diagram

Of the 373 volunteers who were screened by phone, 46 met all inclusion/exclusion criteria. However, after a face to face meeting, 10 were excluded due to refusing to participate further. Therefore, a total of 36 participants completed the study (omega3 group *n* = 18; placebo group n = 18). The mean age of all participants was 21.86 (±3.15) years. The participants were athletes in at last one field (football *n* = 9, volleyball *n* = 3, basketball *n* = 4, athletics *n* = 2, archery n = 1, martial arts *n* = 6, swimming n = 4, weightlifting n = 3, wrestling n = 1, rock climbing n = 3). Table 2 shows the baseline demographic characteristics, blood pressure, and physical activity for intervention and placebo groups. There was no significant difference for any demographic parameter between groups. No adverse effects were reported by any of the participants at any stage of the study. Figure 1 shows the study flow diagram.

**Table 2:** Baseline characteristics of the study participants

Variables	Omega-3 Group (*n* = 18)	Placebo Group (n = 18)	p
**Age (year)**^**a**^	21.8 (3.71)	21.8 (2.59)	0.05^c^
**Education** ^**b**^			
< Bachelor	8 (44.4)	4 (22.2)	0.07^d^
Bachelor Student	10 (55.5)	10 (55.5)	
≥ Bachelor	0 (00.0)	4 (22.2)	
**Marital status** ^**b**^			
Single	15 (83.3)	16 (88.8)	1.00^c^
Married	3 (16.6)	2 (11.1)	
**SBP (mmHg)**^a^	110 (100)	110 (110)	0.96^c^
**DBP (mmHg)**^a^	70 (70.7)	71.5 (70.7)	0.60^c^
**Physical activity level**^b^			
Low and Moderate	8 (44.4)	10 (55.5)	0.74^c^
High	10 (55.5)	8 (44.4)	
**Physical activity (**Met- minute /week)^a^	3143 (3147)	2711 (2782)	0.44^c^
**Weight (Kg)**^a^	73.9 (12.3)	71.8 (13.6)	0.62^c^
**Body fat (percent)**^a^	16.4 (5.27)	15.8 (6.11)	0.76^c^
**FM (kg)**^a^	12.7 (6.31)	12.1 (6.25)	0.76^c^
**FFM (kg)**^a^	61.2 (6.80)	59.7 (7.99)	0.56^c^

^a^Mean (standard deviation). ^b^Frequency (%)

^c^Based on independent-samples t-test. ^d^Based on Chi-Square test

*SBP* Systolic blood pressure, *DBP* Diastolic blood pressure, *Met* Metabolic equivalent of task, *FM* fat mass, *FFM* fat free mass

### Effects of omega3 on the REE

As presented in Table 3, VO2 max, and REE were increased significantly in the omega3 group (*p* < 0.05). Also, REE was increased by 10.67% in the omega3 group (*p* < 0.001), and after baseline adjustments, the increase was 22.83% (*p* < 0.01).

**Table 3:** Comparison of the REE between study groups before and after the intervention

Variable	Omega-3 Group (n = 18)	Placebo Group (n = 18)	MD (95% CI), P
**VO2 max (mL/kg/min)**			
Before	264 (61.1)	219 (57.9)	44.9 (4.62, 85.2), 0.98^b^
After	280 (66.6)	225 (48.9)	54.5, (14.9, 94.1), 0.01^a^
MD (95% CI), p^a^	−15.7 (−29.5, − 1.94), 0.03	−6.17 (−23.3, 11.0), 0.46	
**REE (kcal/day)**			
Before	1792 (311)	1527 (404)	265 (20.4, 509), 0.98^b^
After	2012 (401)	1557 (344)	454 (201, 708), 0.01^c^
MD (95% CI), p^a^	− 220(−341, −98.3), 0.01	−30.6(−181, 120), 0.67	
**REE%**			
Before	99.5 (20.1)	85.3 (22.2)	14.1 (− 0.27, 28.5), 0.05^b^
After	110 (23.1)	87.3 (19.7)	22.8 (8.27, 37.3), 0.01^c^
MD (95% CI), p^a^	−10.6(−16.1, −5.14), 0.01	− 1.94(− 7.79, 3.90), 0.49	

Data are presented as Mean (standard deviation)

^a^ p based on paired samples t-test

^b^ p based on independent-samples t-test

^c^ based on ANCOVA adjusted for baseline values

*CI* confidence of interval, *MD* mean differences, *REE* resting energy expenditure, *VO2 max* Maximal oxygen consumption

### Effects of omega3 on appetite

**Fig. 2:**
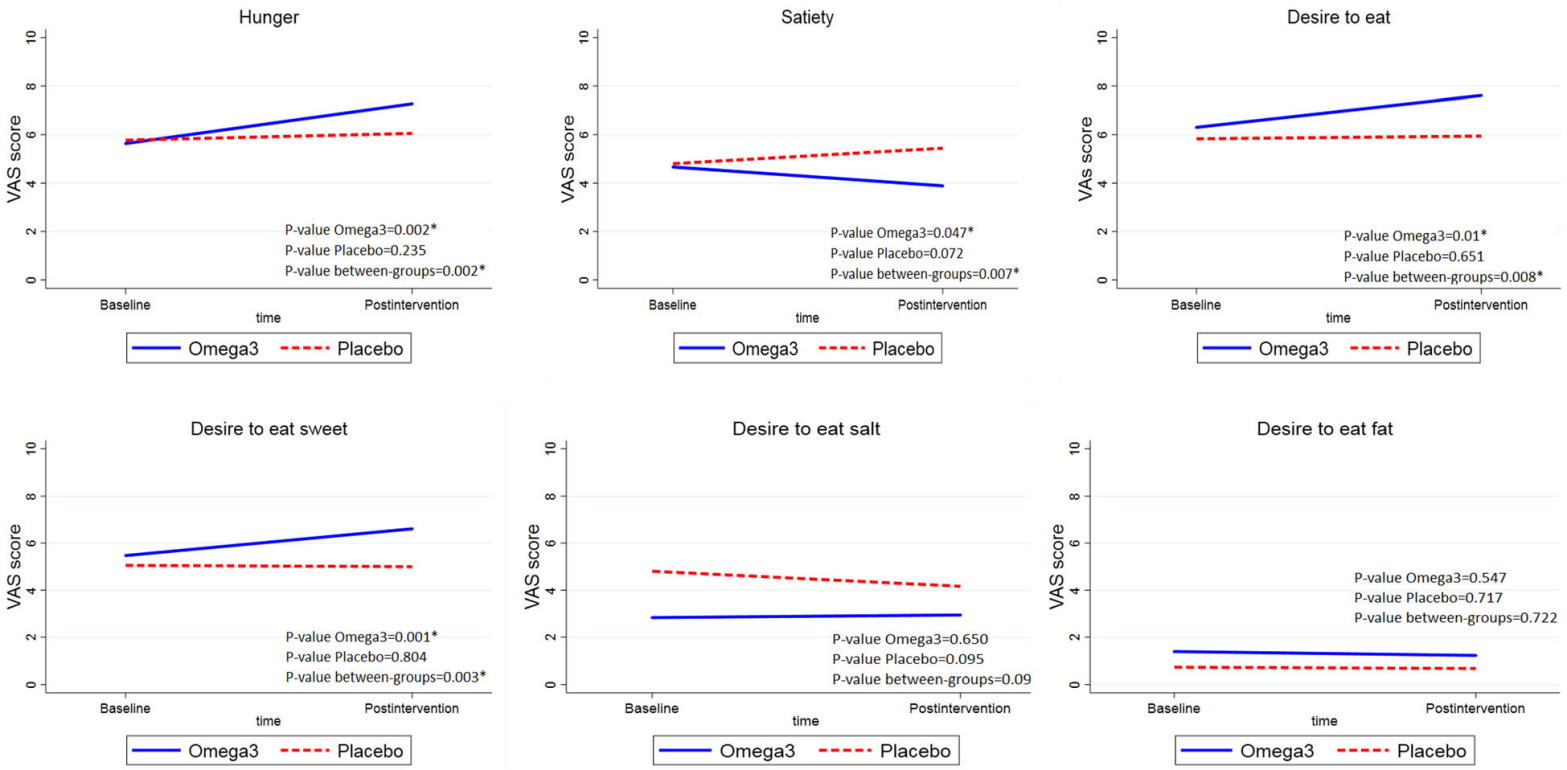
VAS score at baseline and end of the study in omega3 and placebo group. Solid lines reveal intervention group, and dash lines demonstrate placebo group. VAS: Visual analogue scale

Figure 2 presents VAS score at baseline and end of the study in omega3 and placebo groups. After three weeks of omega3 consumption, the sensation of hunger, desire to eat, desire to eat sweet taste, and desire to eat salty foods increased, but satiety and desire to eat fat decreased (*p* < 0.05).

### Effects of omega3 on the UCP2 levels in the blood

Table 4 compares the levels of UCP2 protein between study groups at baseline and the end of the intervention. The amount of UCP2 in blood was increased significantly in the intervention group (MD = 6.48 μg, *p* = 0.001), but decreased in the placebo group by 0.32 μg (*p* = 0.009). Compared to baseline levels, UCP2 increased by 7.17 μg (95% CI = 4.02, 12.85; *P* = 0.04).

**Table 4:** Comparison of the serum concentrations of UCP2 at before and after the intervention

Variable	Omega-3 Group (n = 18)	Placebo Group (n = 18)	MD (95% CI), P
Ucp2 (ng/L)			
Before	15.6 (11.5, 27.9)	15.2 (11.8, 28.1)	0.37(− 0.47, 0.42), 0.91^b^
After	22.1 (13.4, 29.4)	14.9 (10.4, 17.8)	7.17 (4.02, 12.8), 0.04 ^c^
MD (95% CI), p^a^	6.48 (3.08, 7.73), 0.01	−0.32 (0.12, 0.49), 0.01	

*UCP2* uncoupling protein2; Mean (standard deviation), and mean difference (95% CI)

^a^ based on Wilcoxon signed-rank test

^b^ based on Mann–Whitney U test

^c^ based on Quantile regression adjusted for baseline values

### Effects of omega 3 fatty acids on PPARγ and UCP2 gene expression

**Fig. 3:**
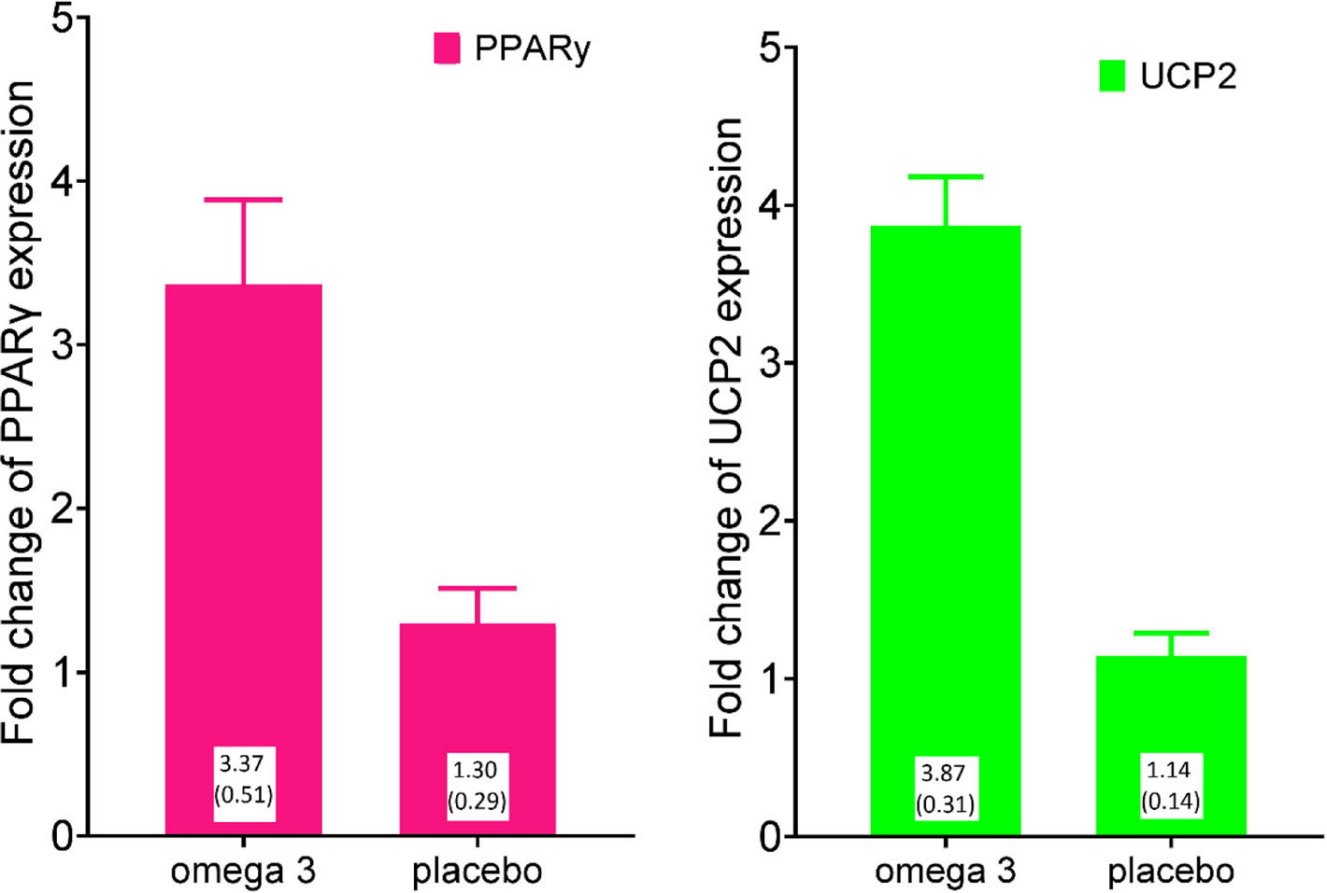
Effect of 3 weeks omega3 supplementation on expression ratio. (p < 0.01 for both diagrams). **a**. Mean fold change of UCP2 expression in omega3 and placebo groups in comparison of GAPDH. **b**. Mean difference in fold change of PPARγ expression in omega3 and placebo groups in comparison of GAPDH. GAPDH: glyceraldehyde-3-phosphate dehydrogenase; UCP2: uncoupling protein2; PPARγ: peroxisome proliferator-activated receptor gamma

The Omega 3 and placebo groups had a significant difference in the fold change of PPARγ gene expression (*p* < 0.05). The mean (SE) fold change expression of UCP2 in comparison to GAPDH, in the Omega 3 group was 3.87 (0.31) and in the placebo group was 1.14 (0.14). Also, the Omega 3 and placebo groups also showed a significant difference in the fold change of PPARγ gene expression (p < 0.05). Mean (SE) fold change expression of in comparison to GAPDH, in the Omega 3 group was 3.37 (0.51), and in the placebo group was 1.30 (0.29). Figure 3 shows the difference between gene expressions of PPARγ and UCP2 in Omega 3 and Placebo groups.

## Discussion

A large body of research has focused on PPARγ as a regulator of energy metabolism. On the other hand, many studies suggest that omega3 improves metabolism and homeostasis through the regulation of genes related to PPARγ signaling but the exact mechanisms were not known [29, 58]. Our results revealed that the level of PPARγ mRNA expression is significantly up-regulated in athletes who were administered omega3 supplements (*p* < 0.05). In line with our data, cell culture and animal studies demonstrate Omega 3 supplementation increased the expression of PPARγ [59–61]. Also, previous human studies showed in patients with obesity, diabetes mellitus, and gestational diabetes mellitus, omega3 supplementation unregulated gene expression of PPARγ [62–64]. A few studies showed omega3 had no significant effects on PPARγ mRNA levels in cultured cells, and even patients with diabetes mellitus [59, 65, 66] and one study indicated that twelve-weeks of omega3 supplementation in adolescents with obesity down-regulated PPARγ mRNA expression [58]. A lack of an effect of omega3 on PPARγ gene expression could be a consequence of a low expression of retinoid X receptor (RXR), as a heterodimer of PPARγ [60]. In light of these studies and our study, it seems that omega3, as a ligand of PPARγ, could up-regulate PPARγ mRNA expression. Further studies may be required to discover the exact molecular basis by which omega3 supplementation affects PPARγ expression.

**Fig. 4:**
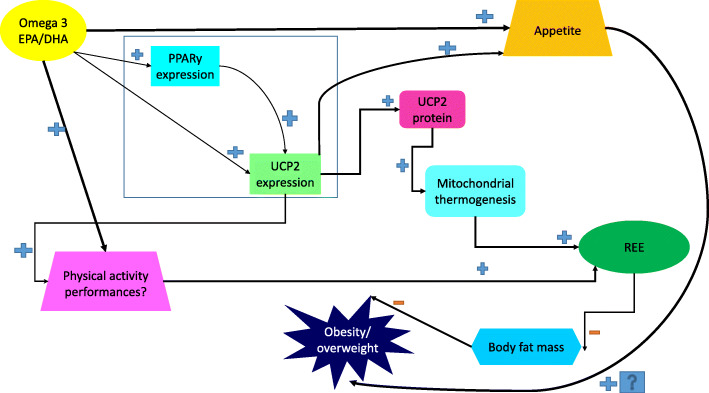
The relation of Omega 3 fatty acids, REE, PPARγ and UCP2. DHA = docosahexaenoic acid; EPA = eicosapentaenoic acid; PPARγ = Peroxisome proliferator-activated receptor-gamma; REE = Resting metabolic rate; UCP2 = Uncoupling protein2

By activating PPARγ, omega3 supplementation may enhance mitochondrial fatty acid oxidation [67]. One of the main promoters of oxidation in mitochondria are uncoupling proteins, especially UCP2 [42]. As presented in this study, after omega3 supplementation, UCP2 mRNA expression increased by 3.85 fold. Many cell culture and rodent studies show EPA or DHA increases UCP2 expression [68–72]. In agreement with our results, fish oil feeding can up-regulate mRNA of the UCP2 mRNA by five fold in mice [73]. Mice fed with omega3-containing food increased UCP2 mRNA in white adipose tissue 2.7 fold [72, 74]. Also, in mammals, UCP2 protein levels increase by the effect of omega3s [37]. In humans, two months of DHA-enriched food in football players increased UCP2 expression, although it was concluded that this enhancement might have been caused by oxidative stress due to exercise [75]. Another study showed eight weeks of DHA supplementation increased UCP2 protein levels after training [76]. In contrast to our study, two animal studies showed DHA and DHA-rich tuna oil did not affect UCP2 expression in rodents [77, 78] and an additional study demonstrated EPA suppressed overexpression of UCP2 in mice [79]. Another study found that after fish oil supplementation UCP2 was actually down-regulated [80]. The reason for these contrasting results could be due to different doses and duration of supplementation, the different conditions in cell cultures, and also the differences in the body components assessed across studies. In this regard, further studies are required to assess the different changes in UCP2 by omega3 supplementation in different health or disease conditions. Figure 4 shows the relation of omega 3 fatty acids, PPARγ and UCP2 in the terms of energy metabolism.

As appetite is regulated by metabolic hormones and neurotransmitters, the genetic factors that affect metabolism (including PPARγ and subsequently UCP2 expression) could affect hunger sensation [43–46]. Barazzoni et al. (2004) showed appetite increased in rodents by increasing in PPARγ mRNA expression [81]. To our knowledge until now no study investigate this relationship in human. However, in our study increased PPARγ is related to increased appetite. Results across studies vary, however, as it has also been shown that increasing PPARγ expression suppresses ghrelin mRNA expression and thus reduces appetite and obesity [47, 48].

Appetite sensations are related to energy balance in the long term and can be affected by dietary factors such as omega3 fatty acids [82]. In our study, omega3 supplementation for three weeks increased hunger sensation (and desire to eat), and also decreased satiety. In agreement with these results, studies with similar duration (three weeks) demonstrated omega3 supplementation decreased satiety or increased hunger with 3.5 g omega3 or a combination of omega3 and omega6 in healthy individuals [49, 83]. However, in contrast to our study, weight loss program in individuals with overweight and obesity imply that omega3s may increase fullness, suppress appetite, or decrease hunger [84]. Differences in study populations, dose, and duration of intervention, physical activity, and nutrient intakes can be considered as reasons for conflicting results.

Our study showed an increase in resting energy expenditure with omega3 supplementation. UCP2 protein has been associated with resting energy expenditure, as subjects with obesity and low level of UCP2 protein have low resting energy expenditure [44]. In healthy humans, the effect of omega3s on REE (i.e. resting metabolic rate) is limited and controversial. In agreement with our study, some have reported REE increased by 5% after fish oil supplementation [85], while others reported no significant effect [86–88].

One study showed omega3 in the rat was associated with increased activity of the sodium-potassium pump ATPase, increased mitochondrial proton leak, and enhanced energy expenditure [89]. Omega3 binds to PPARγ, potentially altering the expression of proteins involved in fat metabolism. In general, it was suggested that supplementation with EPA and DHA may increase REE through enhanced fatty acid oxidation [90]. Some factors that may have contributed to the variable results in previous studies include low doses of omega3, shorter and variable supplementation periods, and small numbers of participants. Figure 4 shows the relation of omega 3 fatty acids, REE, PPARγ and UCP2.

A limitation of our study is the differential effects of EPA and DHA were not assessed. Another limitation is that as PPARγ is a nuclear receptor, it requires ligand for its activation and subsequent nuclear translocation. Compared to its expression levels, its protein level is very important, unfortunately this study could not measure it. The evaluation of expression levels of downstream target genes other than UCP2 is suggested for future research. Future studies should assess the effect of omega3 supplementation on fat distribution in the body, such as visceral or subcutaneous fat, and assess the effect of plant omega3s which are converted to DHA and EPA in the body. It is also recommended to evaluate the effect of very high doses and different durations of omega3s on altering the body composition. Also, we recommend the evaluation of the combination of omega3 with other obesity-related nutrients (especially vitamin D and E, Q10).

## Conclusion

Omega 3 fatty acids may have an important role in affecting energy metabolism. Our findings showed that omega3 supplementation leads to the up-regulation of PPARγ and UCP2 expression in healthy athletes. These data provide additional evidence to support the hypothesis that these genes may act as a potential target for enhancing REE and appetite. Further investigations are suggested to confirm and support the recommendation of omega3s for weight reduction in patients with obesity-comorbidities.

## Data Availability

The datasets used and/or analysed during the current study available from the corresponding author on reasonable request.

## Abbreviations

ANCOVA: Analysis of covariance
BMI: Body mass index
BP: Blood pressure
cDNA: Complementary DNA
DHA: Docosahexaenoic acid
EPA: Eicosapentaenoic acid
FM: fat mass
FFM: fat free mass
GPAC: global physical activity questionnaire
MET: metabolic equivalent of task
PPARγ: Peroxisome proliferator-activated receptor-gamma
PBMC: Peripheral blood mononuclear cell
REE: Resting metabolic rate
UCP2: Uncoupling protein2
VO2 max: Maximal oxygen consumption
